# Age at menarche and performance intelligence quotients of adolescents in Bangkok, Thailand: a cross-sectional study

**DOI:** 10.1186/s12887-016-0624-8

**Published:** 2016-07-11

**Authors:** Pongsak Noipayak, Petch Rawdaree, Busaba Supawattanabodee, Sumonmal Manusirivitthaya

**Affiliations:** Department of Pediatrics, Navamindradhiraj University, Vajira Hospital, Samsean Road, Dusit, Bangkok, 10300 Thailand; Department of Medicine, Navamindradhiraj University, Bangkok, Thailand; Research Promoting Center, Faculty of Medicine, Vajira Hospital, Navamindradhiraj University, Bangkok, Thailand

**Keywords:** Performance IQ, Bangkok, Menarche, Early puberty, Maternal age

## Abstract

**Background:**

The presence of an association between age at the onset of puberty and intelligence quotient (IQ) in young adolescents remains controversial. The aim of this study was to explore the association between age at menarche and performance IQ scores of young female adolescents in Bangkok, Thailand.

**Methods:**

A cross-sectional study was conducted among 537 students aged 11–15 years attending primary and secondary schools in the Dusit district of Bangkok, Thailand. The participants were selected based on two-step stratified sampling. Age at menarche and health and socioeconomic status were determined using a self-report questionnaire completed by participants. Performance IQ scores were determined using the Standard Progressive Matrices intelligence test (Thai version) administered by registered clinical psychologists.

**Results:**

Of the 537 participants, 0.4 had reached menarche at 8 years of age, 1.9 at 9 years, 10.1 at 10 years, 36.1 at 11 years, 37.6 at 12 years, 10.4 at 13 years, 3.4 at 14 years, and 0.2 % at 15 years. Age at menarche was inversely correlated with performance IQ (Pearson correlation −0.087, *p* = 0.047). The regression equation predicting performance IQ by age at menarche was performance IQ = 128.06 − 1.16*age at menarche (*R*^*2*^ = 0.008). In univariate analysis, performance IQ was inversely correlated with age at menarche, body mass index (BMI), time spent watching television, and time sleeping, but was directly correlated with maternal age at birth (all *p* < 0.05). In multivariate analysis, age at menarche and BMI remained significantly inversely correlated with performance IQ (*p* < 0.05), while maternal age at birth was directly correlated with performance IQ. The model consisting of age at menarche, BMI, and maternal age at birth best predicted performance IQ.

**Conclusion:**

After adjusting for confounders, multiple regression analysis showed that age at menarche and BMI of young female adolescents living in the Dusit district of Bangkok, Thailand, were inversely correlated with performance IQ, whereas maternal age at birth was directly correlated with performance IQ.

## Background

The age at menarche has been decreasing worldwide over the last century. The secular trend is approximately 2 months per decade in the United States and 2.5 months per decade in Europe [1]. Two surveys conducted in the United States revealed a decrease in age at menarche from 12.8 years in 1963–1970 to 12.5 years in 1988–1994 [2]. In Asia, a study of adolescent Indian girls found that the age at menarche had decreased by approximately 7 months per decade since 1960 [3]. There have been two previous studies on the mean age at menarche among different population groups in Thailand. In a 1987 study of female adolescents living in Bangkok, the mean age at menarche was 12.15 years [4]. In a 1997 study of obese teenagers living in rural areas of Thailand, the mean age at menarche was 12.5 years [5].

Hormonal changes during adolescence, such as increases in androstenedione, and increases in dehydroepiandrosterone and its sulfate have been reported to be associated not only with pubertal onset but also with the development and function of the central nervous system [6]. However, reports of the association between early puberty and brain function, particularly intelligence quotient (IQ) around the time of menarche, remain inconsistent. In 1983, a study comparing the IQ of girls with early puberty and matched controls found that the girls with early puberty had poorer verbal skills but better spatial abilities than the controls [7]. In contrast, the findings of a study published in 1984 reported that girls with early puberty had higher verbal IQ than control subjects, but there was no difference in the performance IQ [8]. A study on performance IQ among adolescent girls in Iran found that performance IQ was inversely correlated with age at menarche [9], and a Danish study reported in 1986 found that while the long-term IQ of girls with early puberty did not differ from that of control subjects, the spatial perception scores of girls with early puberty were lower [10]. A 2012 study of girls living in the United States compared the IQ of girls around the time of menarche and found that girls with early puberty had a lower IQ than controls [11]. The inconsistent findings of previous studies make it difficult to draw a conclusion about any association between early puberty and IQ scores. The present study aimed to explore the association between age at menarche and performance IQ scores of young female adolescents living in the Dusit district of Bangkok, Thailand.

## Methods

### Study design and participants

This study was conducted as part of the Dusit study project, a cross-sectional study conducted between June and December 2013. The project proposed to investigate the association between age at menarche and various health and socioeconomic issues and demographic factors among female students in grades 5–9 at schools in the Dusit district of Bangkok, Thailand. There were 47 schools in Dusit, with a total of 17,045 female students. The schools were categorized into four groups according to their affiliations with the Bangkok Metropolis (Bangkok Metropolitan Administration [BMA] schools), the Office of the Basic Education Commission of Thailand (schools of the Ministry of Education), universities (teacher-training schools), and the Office of the Private Education Commission (private schools). There were no Thai data available to calculate the sample size for the present study. Therefore, the sample size was calculated based on the mean age at menarche ± standard deviation (SD; 12.62 ± 1.05) for Indian girls as reported in 2009 [3]. A minimum of 848 participants was required but the sample size was increased by 20 % to increase the power and to minimize the possibility of inadequate samples because of non-responders. Finally, 1018 female students were included in the target population. This sample size exceeded the number of participants calculated from a study in Iran (*n* = 90) as being required to investigate the association between age at menarche and IQ (correlation = 0.26) [9].

A two-step sampling procedure was carried out to recruit participants. Two mixed-sex BMA schools, one mixed-sex Ministry of Education school, one mixed-sex teacher-training school, and two girls-only private schools were sampled in the first step using simple random sampling according to the ratios of students. Next, to ensure convenient data collection for the whole Dusit study project, students were sampled as clusters based on their classrooms. Written parental consent was obtained for all students included after sample selection before the data collection, but participants with a history of illness involving sex hormones (e.g., pituitary tumor, steroid administration), and those who were unable to read and/or write were excluded.

### Data collection

Eight pediatricians validated the content of the self-report questionnaire used in the study. After content validation (content validity ratio = 0.75–0.99), one question was modified, and five questions about sexuality were deleted. The questionnaires were then sent to the study students via the research coordinator teachers in each school, and were collected within 1 week. Students completed questions on their age at menarche, health and socioeconomic status (such as their allowance), the amount of time spent sleeping (sleeping time), the amount of time spent playing video games, the amount of time spent watching television, and the amount of time spent reading any reading material. If questionnaires were not returned within 3 days of two telephone calls in the second week after distribution, the participants were defined as non-responders.

To ensure the reliability and validity of anthropometric measurements, weight and height were measured at schools by a trained research nurse following the Centers for Disease Control and Prevention (CDC) standard [12], and BMI was calculated using SPSS version 22 (IBM Corp., Armonk, NY, USA). Performance IQ scores were measured by registered clinical psychologists using the Standard Progressive Matrices intelligence test (Thai version), which is a nonverbal intelligence test [13]. The reliability of the Thai version of the Standard Progressive Matrices was 0.946, as determined among Thai children and youths in 2009 using the Wechsler Intelligence Scale for Children-III as a gold standard [13]. The Research Ethics Committee at the Faculty of Medicine Vajira Hospital, Navamindradhiraj University (University of Bangkok Metropolis), Bangkok, Thailand, approved this study on July 30, 2012.

### Statistical analyses

An assistant researcher carried out verification for relevant factors included in the analysis, such as age at menarche, during data entry and data cleaning. Cases with questionable or unreliable age at menarche were excluded from the analysis. Age at menarche and IQ scores were reported as mean ± SD or geometric means and 95 % confidence intervals (CIs), according to the distribution of the data. Quantitative data were analyzed using an independent *t-*test, the Mann–Whitney *U* test, analysis of variance, or the Kruskal–Wallis test, as appropriate. Correlations between variables were assessed by univariate and multivariate regression analysis. Possible confounders and effect modifiers were addressed using the Mantel–Haenszel test and the test for homogeneity from the extended Mantel–Haenszel test. Stepwise regression analysis was conducted to identify the best model for the correlation. A *p*-value < 0.05 was considered statistically significant.

## Results

Of the 1020 students selected for the study, 537 (52.6 %) had already reached menarche. The mean age at menarche was 11.8 ± 1.0 years. The students who had not reached menarche were excluded from the main analysis, but were used as a comparison group where appropriate. Of the 537 girls who were menstruating at the time of the study, 0.4 % (*n* = 2) had reached menarche at 8 years of age, 1.9 (10) at 9 years, 10.1 (54) at 10 years, 36.1 (194) at 11 years, 37.6 (202) at 12 years, 10.4 (56) at 13 years, 3.4(18) at 14 years, and 0.2 % (1) at 15 years.

**Fig. 1:**
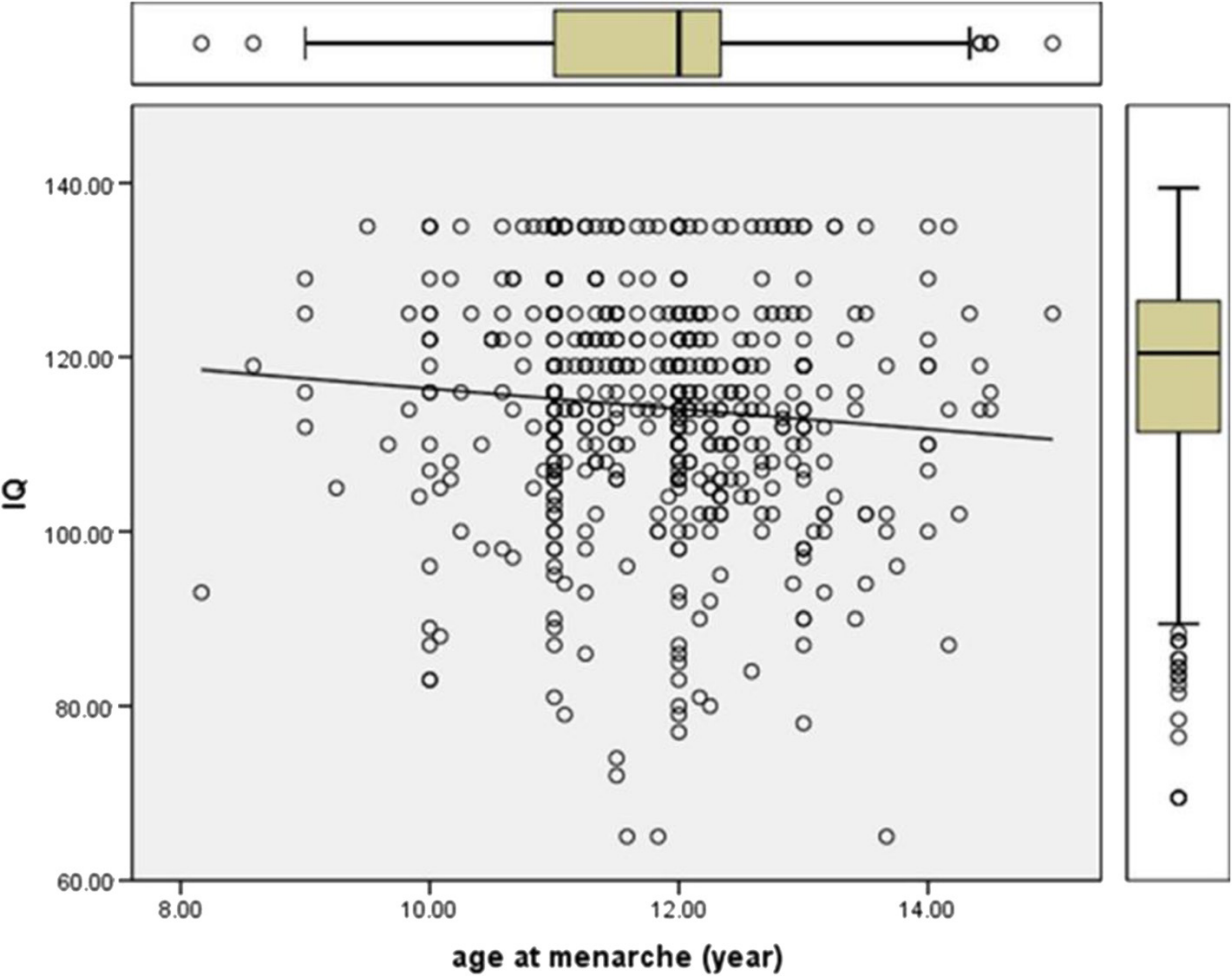
Scatterplot demonstrating the association between performance IQ and age at menarche

The mean performance IQ for all 1020 participants initially selected was 114.3 (95 % CI: 113.4–115.2). The mean performance IQ among the 537 participants who already had their first menstruation at the time of data collection (115.4; 95 % CI: 114.2–116.6) was significantly higher compared with that for the 483 students (47.4 %) who had not reached menarche (113.4; 95 % CI: 112.1–114.6; *p* = 0.023). Subjects who had reached menarche by 11 years of age (*n* = 66) had significantly higher mean performance IQ (116.4; 95 % CI: 113.6–119.4) than those who had their first menstruation after the age of 11 years (*n* = 471; 112.6; 95 % CI: 111.2–113.9; *p* = 0.007). Subset analysis found that participants who had reached menarche at 8 years of age (*n* = 2) and at 9 years of age (*n* = 10) had a mean performance IQ of 105.5 (95 CI: NA) and 117.5 (95 % CI: 109.5–124.5), respectively. The Pearson correlation between performance IQ and age at menarche was performance IQ = 128.06–1.16*age at menarche (*R*^*2*^ = 0.008). There was an inverse association between performance IQ and age at menarche (Fig. 1). In univariate analysis, age at menarche, BMI, time spent watching television, and sleeping time were inversely correlated with performance IQ. These associations were statistically significant (Table 1).

**Table 1:** Univariate regression analysis of factors correlated with performance intelligent quotient (IQ)

Characteristics	Mean ± SD	Range (min–max)	Intercept	Coefficient	Standard error	95 % CI	*p*-value
1. Age at menarche (years)	11.8 ± 1.0	8.2–15.0	128.06	−1.164	0.585	−2.314, −0.014	0.047*
2. BMI (kg/m^2^)	19.4 ± 4.1	10.4–39.1	121.71	−0.339	0.102	−0.539, −0.139	0.001*
3. Maternal age at birth (years)	31.9 ± 5.2	19.0–54.0	104.56	0.241	0.080	0.085, 0.396	0.003*
4. Watching TV (hours)	2.7 ± 2.9	0.0–14.0	116.09	−0.367	0.147	−0.656, −0.078	0.013*
5. Sleeping time (hours)	7.1 ± 1.2	5.0–9.0	121.72	−0.849	0.351	−1.536, −0.160	0.016*

*p ≤ 0.05

Other socioeconomic factors, such as allowance, paternal age at birth, and time spent playing computer games were not associated with performance IQ. In multivariate analysis, age at menarche, BMI, and maternal age at birth were significantly associated with performance IQ (*p* = 0.028, 0.063, and 0.005, respectively), whereas sleeping time and time spent watching television were not associated with performance IQ (*p* = 0.714 and 0.104, respectively). From stepwise multiple regression analysis, a model including age at menarche, BMI, and maternal age at birth was the best-fit model to predict performance IQ (Table 2). Age at menarche and BMI were inversely correlated with performance IQ, whereas maternal age at birth was directly correlated with performance IQ (Table 2).

**Table 2:** Stepwise multiple regression analysis of the best-fit model

IQ	Intercept	Coefficient	Standard error	95 % CI	*p*-value
Age at menarche (years)	119.18	−1.067	0.310	−1.67, −0.459	0.027*
BMI (kg/m^2^)		−0.297	0.108	−0.509, −0.085	0.023*
Maternal age at birth (years)		0.326	0.081	0.167, 0.486	0.005*

*p ≤ 0.05

## Discussion

This cross-sectional study investigated a possible association between age at menarche and performance IQ in young adolescent Thai girls. The findings suggest that there is an effect of early menarche, an important stage in the physical development of female adolescents, on adolescent IQ. The findings agree with those of a study conducted in five educational districts of Mashhad, Iran, which also found that IQ was inversely correlated with age at menarche [9]. However, the present results contradict a number of other studies of precocious puberty, for example, studies from the United States [7, 8, 11] and Denmark [10], which found no difference or a lower IQ among girls with early menarche compared with controls. Several factors may account for these differences, such as genetic factors, experience, or learning processes, and the recruitment of subjects to the studies. The findings of the present study also contradicted several recent reports on the mismatch between physical development and mental maturity [14, 15]. However, IQ is only one part of mental maturity and does not necessarily reflect overall mental maturity. Environmental and/or ethnic factors may also cause IQ to differ among individuals. Furthermore, the test used in the present study, which was selected because it is suitable for a large number of participants, measured only performance IQ. This may represent an important limitation to the present study. The self-reporting of age at menarche is also a potential limitation. An assessment of secondary sex characteristics using the sexual maturity rating should be carried out together with self-reporting age to validate the age of menarche.

This study also found that there were other factors associated with performance IQ, namely BMI and maternal age at birth, which should be explored in greater depth. There was an inverse relationship between BMI and performance IQ, similar to the findings of a systematic review and meta-analysis of four studies among children [16]. A report published in 2014 also found a negative correlation between BMI and IQ among Mexican children [17]. There is evidence that obesity causes a reduction in brain volume, which leads to poor cognitive function [18–20]. However, the single measurement of BMI performed in the present study does not reflect the effect of long-term nutritional status on brain development and function. The direct correlation between maternal age at birth and performance IQ may be related to advantages of older mothers compared with younger mothers in promoting the IQ of their children based on attitude, belief, self-confidence, and socioeconomic status [21–23]. Studies carried out in the Netherlands and Canada also found that maternal age at birth was directly correlated with the IQ of children at 19 years of age [24, 25]. Moreover, another study in the Netherlands, which examined the relationship between maternal age at birth and the apportionment of cultural, economic, and social capital to children, reported a significant positive effect of maternal age at birth on the schooling of children [26]. The reassessment of IQ in young adults, for example, 18 years of age or older, may differentiate between long-term and transitory effects of early menarche on IQ.

## Conclusion

In summary, this study showed that, after adjustment for BMI and maternal age at birth, earlier age at menarche was associated with higher performance IQ in young female adolescents from the Dusit district in Bangkok, Thailand. In addition, obesity may be associated with a lower IQ, and older maternal age at birth with a higher IQ.

## Abbreviations

BMA, Bangkok Metropolitan Administration; BMI, body mass index; CDC, Centers for Disease Control and Prevention; CIs, confidence interval; IQ, intelligence quotient
